# Advancing agriculture with functional NM: “pathways to sustainable and smart farming technologies”

**DOI:** 10.1186/s11671-024-04144-z

**Published:** 2024-12-05

**Authors:** Mir Waqas Alam, Pir Mohammad Junaid, Yonis Gulzar, Buzuayehu Abebe, Mohammed Awad, S. A. Quazi

**Affiliations:** 1 Department of Physics, College of Science, King Faisal University, 31982 Al-Ahsa, Saudi Arabia; 2 Department of Post Harvest Engineering and Technology, Faculty of Agricultural Sciences, Aligarh Muslim University, Aligarh, India; 3 Department of Management Information Systems, College of Business Administration, King Faisal University, 31982 Al-Ahsa, Saudi Arabia; 4 Department of Applied Chemistry, School of Applied Natural Sciences, Adama Science and Technology University, P.O. Box: 1888, Adama, Ethiopia; 5 Department of Chemical Engineering, Toronto Metropolitan University, Toronto, ON Canada; 6 Bapumiya Sirajoddin Patel Arts, Commerce and Science College, Pimpalgaon Kale, Jalgaon Jamod Dist, Buldhana, Maharashtra India

**Keywords:** Nanotechnology in agriculture, Functional NM, Nano-formulated agrochemicals, Precision agriculture, Nano-priming, Crop yield enhancement

## Abstract

The integration of nanotechnology in agriculture offers a transformative approach to improving crop yields, resource efficiency, and ecological sustainability. This review highlights the application of functional NM, such as nano-formulated agrochemicals, nanosensors, and slow-release fertilizers, which enhance the effectiveness of fertilizers and pesticides while minimizing environmental impacts. By leveraging the unique properties of NM, agricultural practices can achieve better nutrient absorption, reduced chemical runoff, and improved water conservation. Innovations like nano-priming can enhance seed germination and drought resilience, while nanosensors enable precise monitoring of soil and crop health. Despite the promising commercial potential, significant challenges persist regarding the safety, ecological impact, and regulatory frameworks for nanomaterial use. This review emphasizes the need for comprehensive safety assessments and standardized risk evaluation protocols to ensure the responsible implementation of nanotechnology in agriculture.

## Highlights

- Functional NM boost crop yield and sustainability in agriculture.
- Nano-priming and hydrogels enhance seed germination, drought resistance, and stress response.
- Nanosensors enable real-time soil and crop health monitoring.
- Ecological risks and regulatory challenges of NM require thorough assessment for safe use.
- These keywords summarize key points on nanotechnology’s benefits in agriculture.

**Graphical Abstract:**

## Graphical abstract

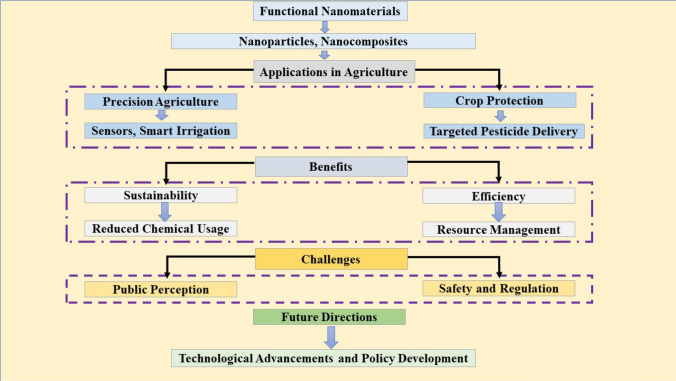

## Introduction

Nanotechnology in agriculture has initiated a revolutionary wave, transforming methods of crop production, pest control, and resource management. Nanotechnology in agriculture has demonstrated its capacity to improve the efficiency and efficacy of conventional farming techniques. Nanomaterials (NM), owing to their diminutive dimensions and high surface area, offer advantages, such as enhanced transportation mechanisms for fertilizers and pesticides, resulting in higher agricultural efficacy and diminished ecological consequences. With the global population projected to reach 9.8 billion by 2050, food production must increase by 70% relative to 2005 levels. Achieving this target requires sustainable intensification of agricultural practices while minimizing the adverse effects, such as soil degradation, eutrophication of water bodies, and greenhouse gas emissions. Nanotechnology offers promising solutions, especially as novel fertilizers that enhance productivity while minimizing environmental harm. The effectiveness of NM as fertilizers is highly variable, contingent upon factors such as soil type, nutrient composition, and the specific characteristics of the nanomaterial employed [1]. Some studies suggest that NM outperform conventional fertilizers, while others find no significant advantage or even inferior results [2–4]. Research on nano-sized fertilizers by Subramanian and Tarafdar shows increased nutrient availability, enhancing plant growth and yield. Nano-pesticides have attracted interest due to their precise, controlled application, leading to effective pest control [5–7]. Marchiol [8] highlighted the role of NM in improving soil fertility and water retention, aiding sustainable water management. Recent studies also explore the impact of nanotechnology on enhancing plant resistance to abiotic stress. El-Saadony et al. [9] demonstrated that nanoparticles boost plants' resilience to climate-induced stressors, thus safeguarding yields.

Smart farming and precision agriculture, driven by nanosensors, enable real-time monitoring of soil and environmental conditions, facilitating data-driven decisions [10, 11]. The shift towards data-driven agriculture facilitates the efficient use of resources and enhances crop management techniques. However, the safety and environmental implications of nanotechnology are critical. Iavicoli and his coworkers presented a thorough analysis of the possible hazards linked to NM, highlighting the importance of conducting rigorous evaluations to guarantee their secure implementation in agricultural activities [12]. The potential for nanotechnological solutions in agriculture to be scaled up and become commercially viable has been widely explored, focusing on both the economic feasibility and the infrastructure required for successful large-scale implementation [13]. This involves considering the sustainability of NM and their lifecycle. The study conducted by Hatami has clarified the beneficial impact of NM on seed vigor and initial plant growth, offering a promising approach to enhance crop yields starting from the germination phase [14]. Some researchers demonstrated that the combination of NM with genetic and microbial technologies has the ability to boost crop resilience and productivity through synergistic effects [15]. Various studies have shown the potential of NM in organic farming [16]. An emerging field that utilizes biotechnological processes to produce diverse nanoscale materials from microorganisms, offering a green alternative to traditional synthetic methods is microbial nanotechnology [17]. This approach enables the creation of biogenic nanomaterials, which are functionalized with bioactive groups, improving their stability and utility in applications ranging from agriculture to environmental remediation.Nanotechnology is set to revolutionize agriculture not only through enhanced crop yields and sustainable practices but also by significantly improving disease management and crop quality. Innovations in NM have enabled the development of advanced sensors capable of detecting bacterial and fungal pathogens at early stages, thereby preventing widespread crop diseases [18, 19].

Despite the promising advancements in nanotechnology, significant research gaps remain. While numerous studies explore the role of NM in enhancing crop productivity and resistance to environmental stressors, there is limited research on their long-term sustainability, scalability, and economic feasibility in real-world agricultural systems. This study seeks to address these gaps by conducting a comprehensive investigation into the practical applications of NM in agriculture, focusing on their effects on crop yield, environmental sustainability, and resource efficiency. Nanotechnology in agriculture involves a comprehensive strategy to improve agricultural output, optimize resource usage, and promote environmental sustainability. The ongoing advancement of this discipline, supported by thorough investigation and creativity, holds the potential for a future in which agriculture becomes not only more efficient and environmentally friendly but also responsive to the intricate challenges of global food security and environmental preservation. Functional NM are used in agriculture to implement many new approaches that aim to improve agricultural productivity, promote environmental sustainability, and optimize resource utilization [20, 21]. The NM, known for their small size and remarkable characteristics, have played a crucial role in transforming agricultural methods.

## Overview of functional NM in agriculture

**Fig. 1:**
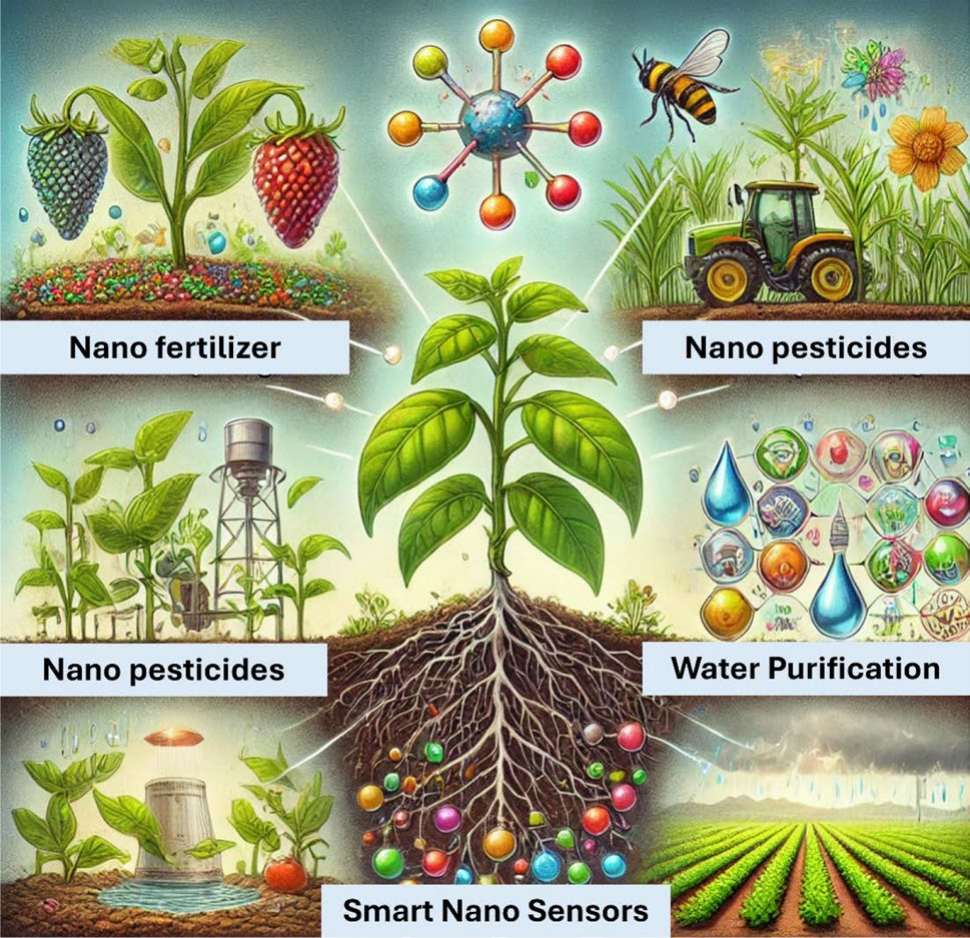
Improvement in the agricultural practices by the implementation of Nanotechnological advancements and diverse applications of nanomaterials, including nano-fertilizers for enhanced plant growth, nano-pesticides for effective pest management, and smart nanosensors for precise monitoring of soil

The emergence of functional NM has revolutionized the agricultural sector, offering innovative solutions to enhance productivity, optimize resource utilization, and promote environmental sustainability. These NM, ranging from nanoparticles to nanocomposites, possess unique physicochemical properties that enable targeted and controlled delivery of agrochemicals, improved nutrient management, and enhanced crop protection [21–23]. NM improve the bioavailability and efficiency of active ingredients, reducing required dosages and minimizing environmental pollution [24]. These materials allow for controlled release and enhanced uptake of essential nutrients, leading to improved plant growth and yield. Nanopesticides provide precise and regulated pest control, reducing risks to non-target organisms and the environment [25]. Nanotechnology significantly enhances the agri-food industry by improving crop productivity through safe and effective nanoagrichemical delivery. Nanotechnology enable early detection of food-borne pathogens and toxins via nanobiotechnological interventions, driven by the synergy between nanotechnology and biosensing technologies [26]. Agricultural nanoproducts, including nano-fertilizers, nano-pesticides, and nano-sensors, are designed to enhance crop productivity and sustainability through precise delivery and targeted action. These nanoproducts are typically synthesized using various methods, including biological processes involving microorganisms, which offer environmentally friendly alternatives to traditional chemical synthesis. For instance, plant extracts and microbial metabolites can facilitate the green synthesis of nanoparticles, ensuring biocompatibility and reduced toxicity. The uptake of these nanomaterials by plants occurs primarily through root absorption and foliar application, where they can penetrate cellular membranes. Molecular mechanisms involved in uptake include endocytosis and passive diffusion, allowing nanoparticles to interact with plant tissues at the cellular level. Once inside, nanoproducts can enhance nutrient availability, improve stress resistance, and enable the targeted delivery of bioactive compounds, ultimately contributing to increased agricultural efficiency and crop resilience. NM in advanced sensing and monitoring systems access real-time, accurate, and continuous monitoring of moisture, nutrient levels, and pathogen presence [27]. This approach of nanosizing integration has resulted in reliable smart agricultural practices. Figure 1: shows the use of Nanotechnologies in different agricultural applications. This vibrant illustration depicts the diverse applications of NM, including nano-fertilizers for enhanced plant growth, nano-pesticides for effective pest management, and smart nanosensors for precise monitoring of soil and water resources [28]. Each component showcases the potential of nanotechnology to revolutionize farming practices by improving efficiency and sustainability."

**Fig. 2:**
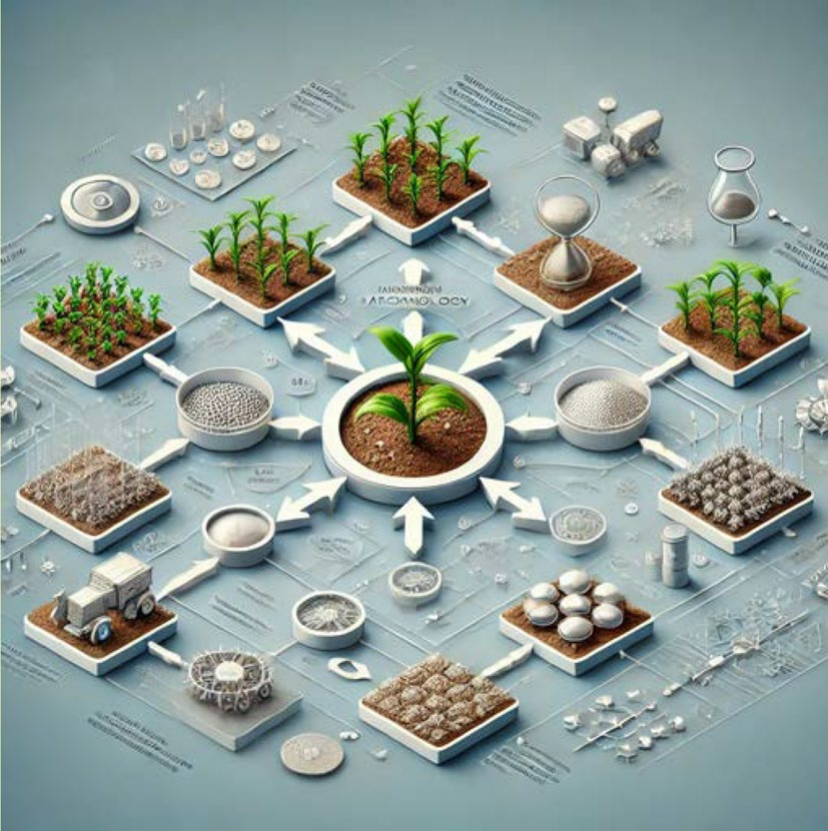
Contribution of Nanotechnology in resilient Agriculture involved in enhancing crop growth, improving soil and water management, and optimizing agricultural outputs through advanced nanotechnologies

The addition of functional NM extends to smart delivery systems contributing to sustainable agriculture by promoting water conservation, reducing chemical runoff, and mitigating environmental footprints. Development of nanofertilizers prioritizes optimizing controlled-release properties, tailoring applications to various agricultural conditions, and addressing regulatory and safety issues to ensure their sustainable use in agriculture [29]. The potential for these materials to transform traditional agricultural practices is ever-increasing, paving the way for a more efficient, environmentally responsible, and productive future in agriculture. Figure 2 shows a comprehensive overview of nanotechnology in agriculture: This detailed flowchart illustrates the integration and applications of various NM in agriculture. It highlights the processes and elements involved in enhancing crop growth, improving soil and water management, and optimizing agricultural outputs through advanced nanotechnologies."

### Nanofertilizers for fertilizers

**Fig. 3:**
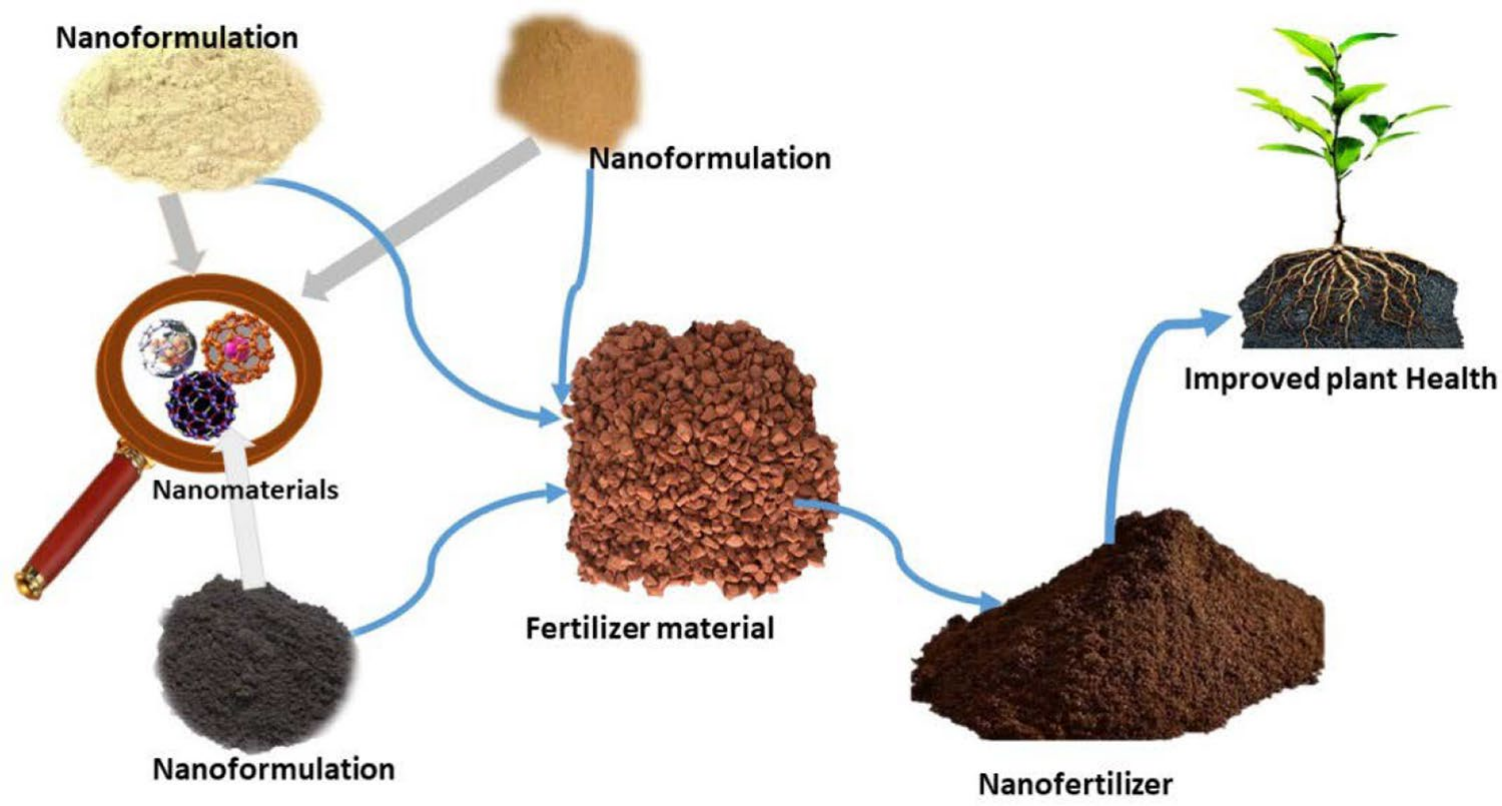
Nanoformulated fertilizer for efficient transport and utilization of essential nutrients required for crop Health

The introduction of nanofertilizers in agriculture represents a significant advancement aimed at addressing the limitations of traditional fertilization practices as shown in Fig. 3. Nanofertilizers enable the efficient transport and utilization of essential nutrients required for crop improvement and growth [30]. These fertilizers provide a controlled, gradual, and consistent release of nutrients at the nanoscale, ensuring a more targeted delivery to plant systems [31]. Nanofertilizers have been shown to enhance the bioavailability and efficiency of critical nutrients such as nitrogen, phosphorus, and potassium, contributing to improved nutrient uptake by plants and minimizing environmental losses through leaching or runoff [32]. Research has shown that NM such as hydroxyapatite nanoparticles (HA-NPs) and layered double hydroxides (LDHs) can serve as effective carriers for nutrients like phosphorus and zinc. These NM have demonstrated potential for controlled nutrient release, which not only matches plant nutrient demands more closely but also reduces the environmental impact associated with traditional fertilizers [33]. HA-NPs have been found to provide a sustained release of phosphorus, improving its availability to plants, particularly in acidic soils where conventional phosphate fertilizers might be less effective due to rapid fixation and low mobility [34]. Similarly, zinc oxide nanoparticles (ZnO-NPs) have been explored for their effectiveness in providing a steady supply of zinc to crops, which is crucial for enzymatic activities and protein synthesis in plants. Studies indicate that ZnO-NPs may enhance zinc content in plant tissues when applied foliar, although the benefits are influenced by factors such as soil type and the nanomaterial's specific properties [35, 36]. Moreover, the use of Zn-loaded NM, such as Zn-complexed chitosan nanoparticles, has shown potential for improving zinc bioavailability and uptake, thereby addressing zinc deficiency in crops grown in zinc-deficient soils [37]. The use of other NM, such as magnesium and iron nanofertilizers, has also been investigated, albeit with mixed results. While some studies report enhanced nutrient availability and crop yields with the use of these NM, others highlight the need for more comprehensive research to fully understand the mechanisms of action and optimize their application in different soil types and agricultural settings. In addition to enhancing nutrient uptake efficiency, the slow and sustained release properties of nanofertilizers help maintain a continuous supply of nutrients to plants, fostering healthier and more resilient crops. As research continues to evolve, the application of NM in fertilizers shows immense promise for advancing sustainable agricultural practices and enhancing global food security. Future studies should focus on refining the formulation of nanofertilizers, optimizing their release kinetics, and evaluating their long-term impacts on soil health and ecosystem sustainability.

The introduction of nanofertilizers in the field of agriculture has been a breakthrough in mitigating the longevity of traditional practices. Nanofertilizers facilitate the transportation and utilization of various essential nutrients for crop improvement and growth as shown in Fig. 1. Nano fertilizers offer the gradual and consistent release of nutrients at the nanoscale warp up [31]. These nano-based fertilizers enhance the bioavailability and efficiency of essential nutrients such as nitrogen, phosphorus, and potassium [38]. These studies provide a clear understanding about the efficiency of nutrient absorption through naotechnological advancements and reduction of environmental losses which are either caused due to leaching or runoff. The slow and sustained release of nutrients from nanofertilizers ensures a steady supply to plants, promoting healthier and more resilient crops. As research in this field progresses, the application of NM in fertilizers holds immense potential for advancing sustainable agricultural practices and enhancing food security globally.

### Nanomaterials for pesticides

**Fig. 4:**
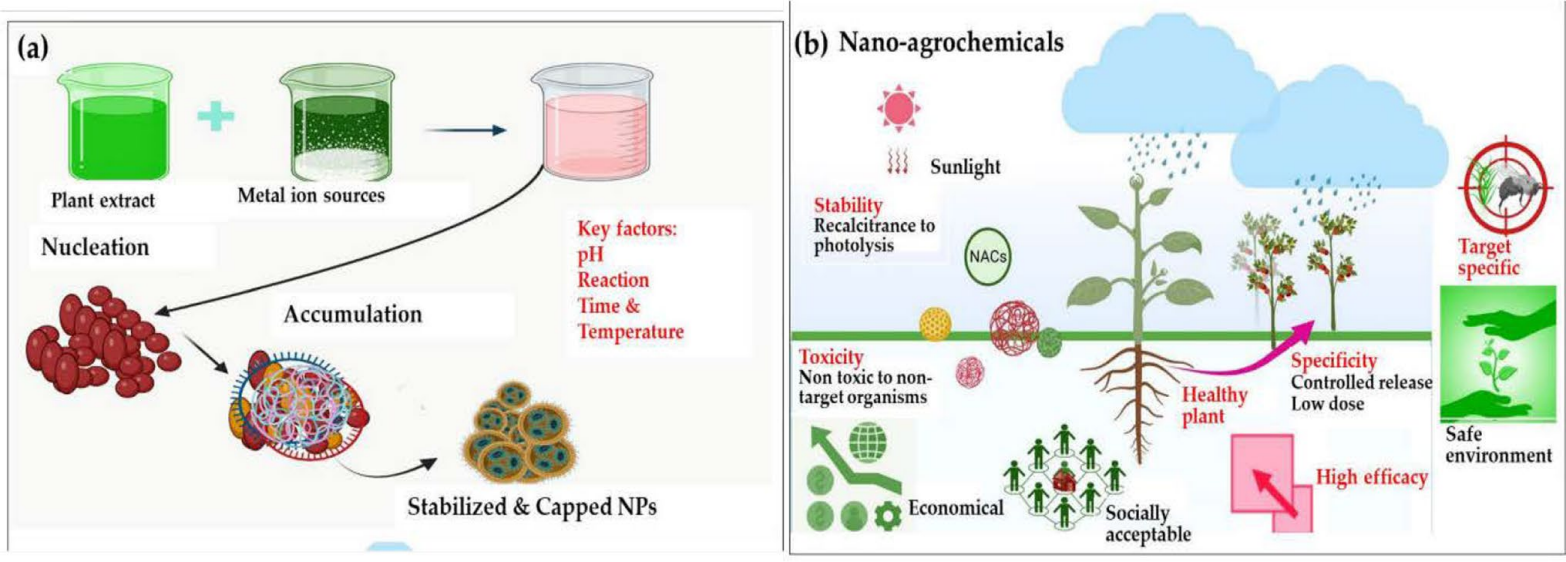
Schematic diagram for the **a** green synthesis of nanoparticles, **b** application of nano-agrochemicals as nanoparticals and improvement of the efficacy of synthetic pesticides: Copy right Ref: [43]

NM are increasingly being used in the development of novel pesticide formulations. Tremendous progress in the field of nanopesticides has been achieved by the use of functional NM. These products provide specific pest control strategies that can decrease the overall amount of chemicals in the environment. At the nanoscale, materials exhibit unique physical, chemical, and biological properties that differ from their bulk counterparts. This allows for the design of more targeted and controlled pesticide release, improved solubility and bioavailability, and enhanced efficacy against pests [39]. Nanocarriers, nanoparticles, and nanoemulsions are some of the prime examples of nanomaterial-enabled pesticide technologies currently being studied and developed [40, 41]. The unique physical features of NM with their precise targeting and release kinetics enable the transformation of agricultural practices.These nanomaterial-based pesticides aim to increase the potency, selectivity, and environmental compatibility of plant protection products. Research conducted by Kumar and his coworkers indicates that nanopesticides are engineered to selectively target particular pests or diseases, hence limiting harm to beneficial organisms and reducing the likelihood of resistance development [42]. Figure 4 shows the eco-friendly green synthesis of nano-agrochemicals (NACs) and their strategic applications in agriculture. The research accentuates utilizing plant extracts, enzymes, and microorganisms to control the synthesis of NM, optimizing their size and shape while minimizing the use of hazardous substances. This method involves plant extracts acting as reducing agents to convert metal ions into stable nanoparticles under controlled conditions like temperature and pH. These nanoparticles are further enhanced by adding functional groups to improve properties such as stability and targeting ability. Part (a) of the image visually details this synthesis process, while part (b) highlights their applications in fields such as pest control, fertilization, and environmental safety. These NM offer targeted action to protect beneficial organisms and reduce environmental impact, illustrating a significant shift towards sustainable agricultural practices (Ali et al. [43] MDPI).

### NM for soil management

Soil health and productivity are the key parameters that determine the effectiveness of agricultural practice. NM have found an increasing scope in retaining soil health and productivity [44]. The nanoscale interaction of the materials with the soil components alters soil properties altogether. NM are used to remediate contaminated soils, removing heavy metals, pesticides, and other pollutants through adsorption or catalytic degradation [45]. Nanoclays and carbon nanotubes have shown promise in improving soil structure, water-holding capacity, and cation exchange capacity [46]. The use of Nano-clays and nano-zeolites demonstrates the ability of soil texture improvement and water retention, hence facilitating superior root growth and plant development [47]. Both the researchers demonstrate significant improvement in soil structure, water retention, and cation exchange capacity, which in turn promotes better root growth and contributes to healthier plant development. Aparicio and his coworkers explore the integration of NM into the soil matrix to enhance aeration, nutrient retention, and microbial activity, resulting in improved soil ecosystems that are healthier and more productive [48]. The property of NM to carry and deliver beneficial microbes, enzymes, and other soil amendments in a targeted manner boosts soil health considerably. The integration of nanotechnology into soil management practices holds significant potential to boost agricultural sustainability, prevent soil degradation, and enhance overall soil health.

### Nanomaterials in water management

The technological advancements in water purification and water treatment have addressed the challenges of global water crises [49]. Advances in material science for the development of nanostructure have prompted in the reduction of water issues. Engineered NM are efficient alternatives for water purification due to their exceptional physicochemical properties [50]. The efficacy of capturing capture heavy metals, organic pollutants, and pathogens through their large surface areas and binding affinities multifold their approach for water purification [51–53]. Panahi demonstrates the efficacy of nano-filters and nano-absorbents in eliminating water contaminants, hence ensuring the presence of clean water for irrigation and decreasing the occurrence of waterborne diseases that impact plants [54]. In comparison to conventional filtration, Nanomembranes and nanostructured filtration mediums achieve higher flux, selectivity, and anti-fouling performance by utilizing precise pore sizes and surface characteristics [55]. Photocatalytic NM power sophisticated oxidation processes to degrade organic pollutants, whereas metal–organic framework nanoparticles enable resource recovery from water sources [56, 57]. The studies collectively highlight the vast potential of nanotechnology in water purification but vary in their specific focuses ranging from pollutant removal and filtration efficiency to advanced degradation processes and resource recovery. The application of nanotechnology demonstrates clear advantages over conventional methods in terms of performance, versatility, and functionality across different environmental and agricultural contexts.

**Fig. 5:**
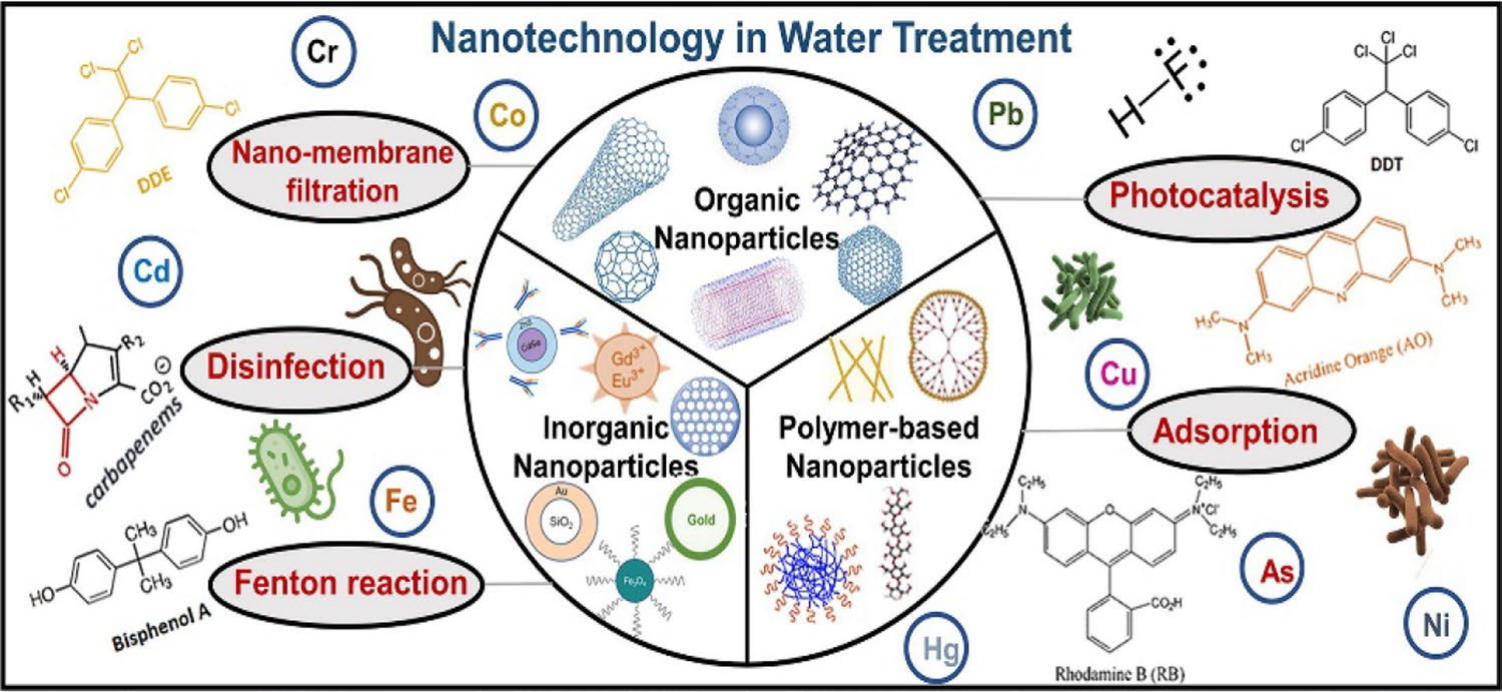
Nanotechnology in water treatment with key techiniques like Nano-membrane filtration, Disinfection, Inorganic Nanoparticles, Organic Nanoparticles, Polymer-based Nanoparticles, Fenton reaction, Photocatalysis, and Adsorption: Copyright ref: [58]

Nanomaterial coatings enhance corrosion resistance and self-cleaning properties in water infrastructure, while nanomaterial-based sensors enable real-time water quality monitoring. The application of nanotechnology in water treatment shows significant promise for improving efficiency and environmental compatibility. Key methods include nano-membrane filtration, disinfection with titanium dioxide, and adsorption using carbon-based materials as shown in Fig. 5. Inorganic nanoparticles, such as gold and silver, facilitate chemical reactions to eliminate contaminants, alongside advanced oxidation processes like the Fenton reaction for breaking down organic pollutants. Overall, nanotechnology offers precise and efficient solutions to complex water treatment challenges, contributing to cleaner and safer water.

### Nanostructure for crop resilience enhancement

Nanostructures have emerged as an appropriate approach for increasing crop resilience and yield in the face of environmental difficulties. At the nanoscale, materials are designed with unique physical, chemical, and biological features that can be used to promote plant health and stress tolerance. Functional NM are currently leading the way in agricultural innovation by providing solutions that improve the efficiency and sustainability of farming processes. Ongoing research and development in the field of nanotechnology in agriculture are continuously revealing new uses and advantages, which hold the potential to improve food security and environmental health in the future. Functional NM are used to improve the ability of crops to withstand environmental challenges like drought, salt, and severe temperatures [59]. Nanoparticles such as silicon dioxide and titanium dioxide have demonstrated the ability to alleviate the impact of stress on plants, resulting in enhanced survival rates and productivity in unfavorable climatic conditions [9, 60]. NM are used to deliver targeted nutrients, pesticides, or genetic materials directly to plant cells, improving nutrient uptake and pest/pathogen resistance [61]. The study conducted by Thompson and Rai has shown that these nanoparticles can activate stress tolerance mechanisms in plants, such as increased antioxidant activity and osmotic adjustment [62]. Improvement in the water retention, UV protection and gas exchange by the leaves and seeds of plant are considerably altered by the application of Nanostructured coatings [63]. The studies collectively reinforce the broad utility of nanotechnology in agriculture, particularly in enhancing stress tolerance, improving nutrient and pesticide delivery, and augmenting physical plant characteristics. However, they diverge in their focus on specific nanomaterials, stress types, and mechanisms of action ranging from biochemical pathways like antioxidant activation to physical enhancements like UV protection and water retention. These differences highlight the multifaceted applications of nanotechnology in agriculture, each contributing to improved plant resilience and productivity from distinct angles.

**Fig. 6:**
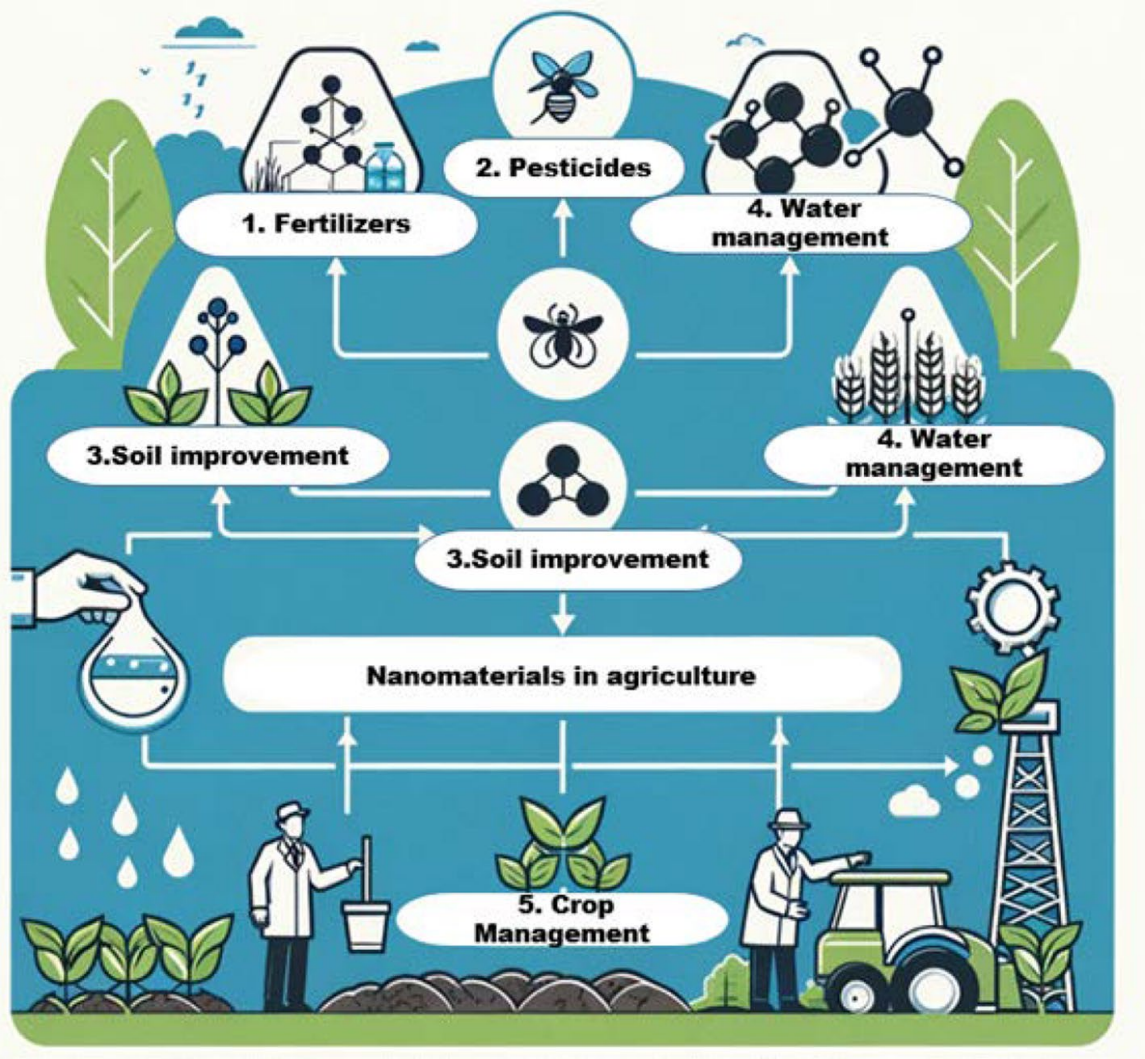
Application of NM in agriculture in key areas such as fertilization, pest control, soil and water management, and enhancing crop resilience:

The ability of the nanostructures to respond to the very small environment changes makes them potential candidates for smart agriculture. As climate change increasingly threatens global food security, nanostructure-enabled crop resilience strategies hold great potential to sustainably boost agricultural productivity. Figure 6 shows the structured overview of how NM are being utilized to advance agricultural practices, focusing on key areas such as fertilization, pest control, soil and water management, and enhancing crop resilience.

## NM for crop protection and disease management

NM hold immense potential to revolutionize crop protection and disease management in agriculture. By targeting specific sites within plants or pests, NM deliver pesticides, fertilizers, and other agricultural chemicals with unparalleled efficiency and precision, reducing the overall quantity of chemicals required and minimizing environmental impact [64]. The controlled release capabilities of NM further enhance their efficacy, ensuring prolonged protection and efficient utilization of active ingredients [65]. This holds a substantial impact on crop protection and disease control, providing innovative, precise, and effective solutions in disease management, NM offer cutting-edge solutions, enabling rapid diagnostic capabilities and the development of novel antimicrobial agents [66]. This precision agriculture approach not only improves crop yields and quality but also promotes sustainable farming practices by lowering chemical inputs and mitigating adverse ecological effects, addressing some of the most pressing challenges faced by modern agriculture as the global population grows and climate change impacts intensify.

### Precision pest management

The start of nanotechnology has led precision pest management, revolutionizing crop protection ways and combating agricultural pests. NM offer unprecedented capabilities for targeted delivery of pesticides and other crop protection agents. The precision targeting of pests and pathogens is facilitated by the application of nanocarriers loaded with active ingredients [67, 68]. The use of nanoencapsulation technique minimizes the damage to beneficial organisms and the environment. These intelligent nanostructures are designed to release their payload in response to specific environmental cues, ensuring optimal timing and dosage for maximum efficacy [68, 69]. Acharya and Pal provide valuable insights into how nano-encapsulated pesticides enhance the precision and effectiveness of pest control measures, thus promoting the sustainability of agricultural practices [70]. The naoencapsulated formulation enables provides a breakthrough in efficient use of pesticides with precision targeting. NM control the detection and monitoring of pest infestations, enabling early intervention and more effective management strategies. To overcome the evolving pests and changing climatic conditions in agricultural sector, nanotechnology holds the promise of a more sustainable, eco-friendly, and productive future for global food production.

### Disease management

The application of NM has emerged as a promising frontier in the management of crop diseases. These structures are designed to enhance the detection, treatment, and prevention of devastating plant pathogens [26]. NM aid in disease management by enabling the precise delivery of fungicides, bactericides, and virucides to specific locations, assuring optimal concentration of these agents [71, 72]. Nanoparticles are designed to act as rapid, sensitive biosensors, enabling early diagnosis of incipient diseases through the identification of specific biomarkers [73–75]. They are utilized as targeted delivery systems, transporting antimicrobial agents directly to affected plant tissues, improving the efficacy of disease control while minimizing the environmental footprint of traditional broad-spectrum treatments [76]. Some nanostructures have even demonstrated intrinsic antifungal or antibacterial properties, providing an additional layer of defense against phytopathogens [77–79]. Gumber and Sidhu [80], highlited the efficacy of nano-formulated fungicides in managing plant diseases, highlighting their improved performance in terms of plant absorption and prolonged release compared to conventional treatments. The application of nanomaterials (NM) in crop disease management has shown significant promise, enabling precise delivery of antimicrobial agents, acting as sensitive biosensors for early disease detection, and demonstrating intrinsic antimicrobial properties.

### Fortifying plant immunity

The integration of NM into agricultural practices holds immense potential for fortifying plant immunity and improving overall crop resilience. At the nanoscale, these engineered structures are precisely tailored to interact with plant cells and tissues, enhancing the innate defense mechanisms of crops. Certain nanoparticles have been found to stimulate the production of phytohormones and other signaling molecules that activate systemic acquired resistance, priming plants to mount a more robust immune response against pathogens and pests [81]. Additionally, NM are used to deliver genetic material or elicitor compounds that trigger the upregulation of genes involved in plant defense pathways [82]. By strengthening the plants' ability to recognize, respond, and adapt to biotic and abiotic stresses, NM offer a transformative approach to sustainable crop protection [9]. By eliciting immunological responses or supplying vital nutrients, they bolster plant resistance to infections. The study conducted by Dong and his coworkers, demonstrates the capacity of NM to stimulate systemic acquired resistance in plants, providing a proactive approach to plant health management [83]. The integration of NM into agriculture offers a transformative approach to crop protection by enhancing plant immunity, stimulating defense mechanisms, and improving resilience against both biotic and abiotic stresses. Nanomaterial-based solutions offer a forward-thinking strategy to combat climate change and emerging disease threats by cultivating a new generation of crops with enhanced immunity and resilience, better equipped to withstand environmental pressures.

### Integrated pest management (IPM)

Integrated Pest Management (IPM) has long been recognized as a comprehensive, environmentally friendly approach to controlling agricultural pests and diseases [84]. The integration of NM into IPM strategies has further amplified the effectiveness and sustainability of these practices [85]. Nanoscale structures serve as smart delivery vehicles, transporting precisely targeted pesticides, biopesticides, or other active ingredients to their intended targets with minimal off-target effects [86, 87]. The integration of nanotechnology into IPM offers a holistic approach that blends NM with traditional approaches for controlling pests and diseases. This comprehensive approach enables the creation of sustainable, efficient, and ecologically conscious methods for protecting crops. By enhancing the specificity and controlled release of these inputs, NM reduce the overall chemical load in the agroecosystem, mitigating the risks of environmental contamination and development of pest resistance [88]. Nanoparticles are designed to serve as biosensors, monitoring pest populations and plant health status in real-time, enabling early detection and timely intervention [89, 90]. This data-driven approach to pest management, combined with the judicious use of eco-friendly control methods, empowers farmers to adopt a more holistic, adaptive IPM framework [91]. Singh and his team examined the incorporation of nanotechnology into IPM, emphasizing its capacity to enhance the effectiveness and durability of pest and disease control in agriculture [92]. NM are essential for the advancement of techniques in crop protection and disease management. Their capacity to deliver focused, effective, and enduring solutions is revolutionizing the field of agricultural pest and disease management. The integration of nanotechnology into IPM enhances the effectiveness and sustainability of pest control practices, offering a more precise and environmentally friendly approach. By combining nanoscale innovations with traditional methods, farmers can achieve a holistic, data-driven strategy that reduces chemical use and minimizes environmental impact while improving crop protection. As the agricultural sector navigates the challenges of climate change and evolving pest threats, the integration of nanomaterial-based technologies into IPM strategies holds the promise of a more resilient, sustainable, and productive future for global food production.

### Smart sensors for precision agriculture

**Fig. 7:**
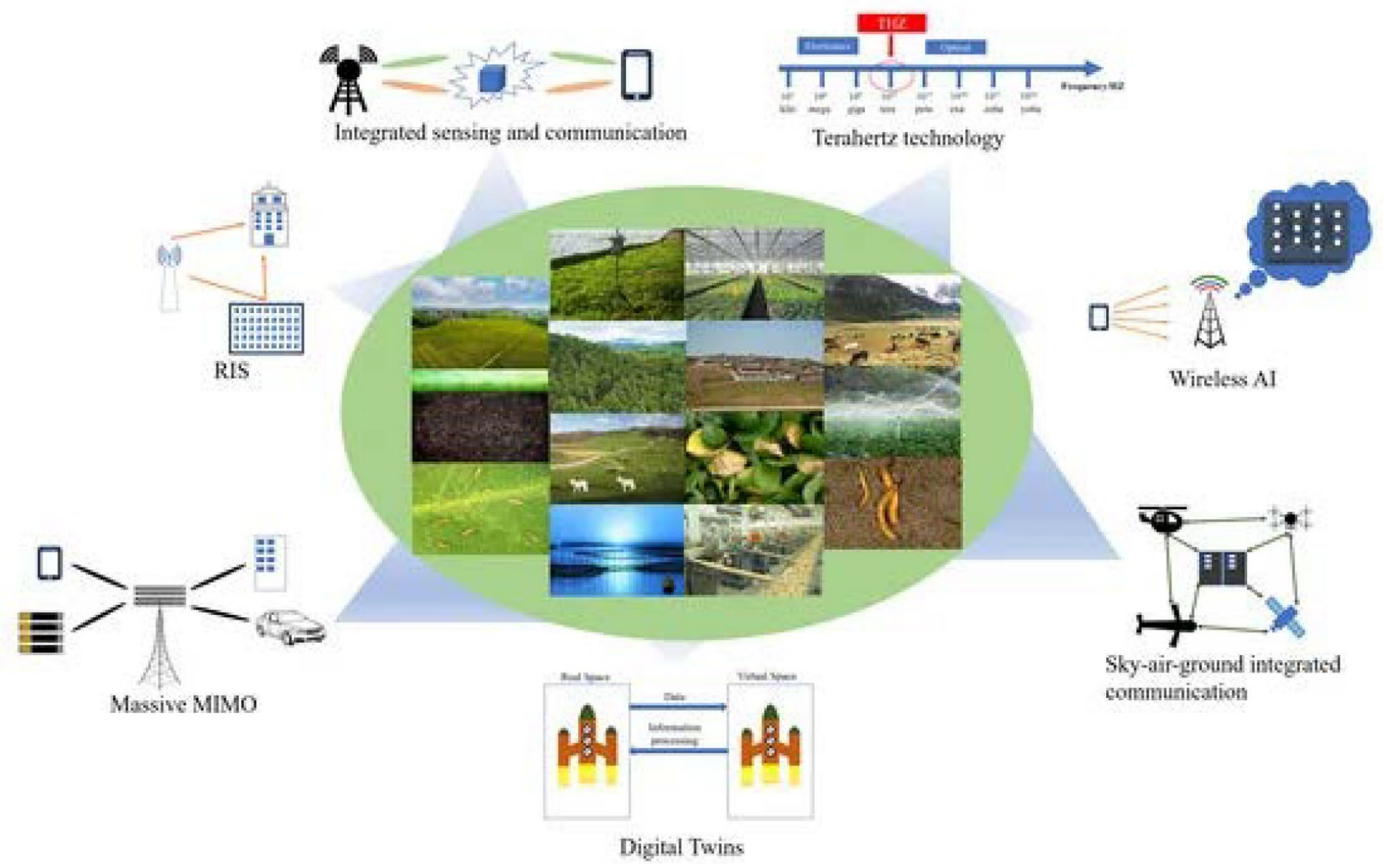
Diverse technologies such as connectivity, intelligence, and sensing are associated with 6G that are set to revolutionize the agricultural sector. Copyright ref: [98]

Smart sensors, which employ nanotechnology, have completely transformed the method by which farmers observe the well-being of crops and the surrounding environmental circumstances. These sensors can identify several aspects, including soil moisture, nutrient levels, insect presence, and disease symptoms serving crucial for making prompt and precise decisions [93–96]. The incorporation of intelligent sensors in agriculture facilitates more accurate and focused interventions, hence decreasing the necessity for wide-ranging pesticide applications and fostering the promotion of healthier crop development [97]. Studies conducted in this field, such as the research conducted by Hashim and his coworkers, illustrate the successful utilization of nanosensor technology for the identification of pathogens and stress conditions in plants [97]. This advancement not only enhances decision-making but also supports sustainable agricultural practices by effectively identifying pathogens and stress conditions in plants. Table 1 provides a concise overview of the effects of nanotechnology on several facets of agriculture. The text classifies the impacts of NM, including nanopesticides, nanoherbicides, and nanofertilizers, on pest control, weed management, and crop yield improvement. Additionally, it demonstrates the exact percentage enhancements in the management of pests and weeds, the growth of crop yields, and the decrease in the use of chemicals as a result of the implementation of nanotechnology. This quantitative analysis offers a concise overview of the extent to which NM enhance agricultural practices by increasing efficiency, sustainability, and productivity. Figure 7 shows the diverse technologies associated with 6G that are set to revolutionize the agricultural sector. As 5G transitions into mass commercialization, its limitations such as high infrastructure costs, limited coverage, and security concerns in software-defined networks are being addressed by advancing into 6G. This next generation promises a new era of connectivity, intelligence, and sensing, driving a comprehensive digital transformation across industries. Specifically for agriculture, 6G will enhance integrated sensing and communication, utilize terahertz technology for faster data transmission, leverage wireless artificial intelligence, and incorporate massive Multiple Input Multiple Output (MIMO) systems for improved signal and coverage. Other innovations shown include sky-air-ground integrated communication systems, digital twins for virtual simulation and management of farming processes, and reflective intelligent surfaces (RIS) that optimize wireless communication efficiency. Collectively, these technologies are poised to significantly advance smart agriculture, integrating digital, physical, and biological systems more seamlessly, and fostering a robust environment for the agricultural industry to flourish in the digital age.

**Table 1:** Impact of nanotechnology on crop protection and disease management

Nanotechnology application	Impact on crop protection	Percentage improvement	Notes	References
Nanopesticides	Reduction in pest population	Up to 60%	Targeted pest control with less environmental impact	[99]
Nanoherbicides	Weed control efficiency	Up to 50%	Precise application reduces non-target effects	[100]
Nanofertilizers	Increase in crop yield	Up to 30%	Enhanced nutrient uptake and reduced fertilizer runoff	[101]
Smart Nanosensors	Improvement in disease detection	Significant	Early detection allows for timely management of plant diseases	[102]
Nanoparticles for plant immunity	Enhanced resistance to pathogens	Varied	Depends on crop type and nanoparticle used	[103]

## NM for soil and water management

The application of NM has opened up transformative possibilities in the safeguarde of soil and water resources within the agricultural sector. The NM are designed to address various challenges, from enhancing soil fertility to improving water quality and utilization. The precision delivery of fertilizers, micronutrients, and soil amendments, has ensured optimal nutrient uptake and minimizing losses through leaching or runoff [4, 104]. Some NM have even demonstrated the ability to sequester heavy metals, pesticide residues, and other contaminants from soil and water, purifying these vital resources [105–107]. NM offer the possibilities to develop smart irrigation systems and water-repellent coatings that maximize water-use efficiency [108]. By integrating these cutting-edge nanomaterial-based technologies into comprehensive soil and water management strategies, farmers cultivate healthier, more productive agroecosystems that are better equipped to withstand the impacts of climate change and environmental pressures. As global demands on natural resources escalate, the transformative potential of NM in sustainable agriculture cannot be overstated.

### Improving soil quality

Integrating NM into agricultural practices holds tremendous promise for sustainably improving soil quality and fertility. Nanoparticles enhance the availability and uptake of essential nutrients by plant roots, addressing deficiencies and imbalances in the soil [109]. Some NM have even demonstrated the ability to sequester heavy metals and other contaminants, remediating degraded soils and mitigating the risks of toxicity [110, 111]. Certain nanostructures are utilized as carriers for beneficial microorganisms, such as nitrogen-fixing bacteria and mycorrhizal fungi, fostering the development of thriving soil biomes that support plant health and productivity [112, 113]. By tailoring the physical, chemical, and biological properties of NM, researchers are developing innovative solutions to revitalize depleted soils, improve soil structure and water-holding capacity, and promote the cycling of organic matter—all of which are crucial for sustainable crop cultivation [114, 115]. As the agricultural sector confronts the challenges of land degradation and the need for more efficient resource utilization, the strategic deployment of nanomaterial-based technologies helps cultivate healthy, resilient soils that underpin the long-term viability of global food production. NM, including nano-clays, nano-zeolites, and carbon-based NM, have demonstrated potential in improving soil structure, water retention, and nutrient availability in soil management [116–118]. NM can enhance soil aeration, augment water-holding capacity, and promote the gradual release of nutrients resulting in enhanced crop growth and improved soil health [119]. Various studies have shown that nano-clays have the ability to greatly improve the texture and fertility of soil, leading to improved root formation and plant growth [120]. NM are being progressively acknowledged for their capacity to completely transform soil and water management in agriculture, by effectively tackling pressing issues such as soil degradation, water scarcity, and inefficient nutrient utilization.

### NM for management and preservation of water resources

The careful management and preservation of water resources are of paramount importance for the long-term sustainability of global agriculture. NM have emerged as transformative tools in addressing this critical challenge. Nanoparticles are engineered to purify and remediate water sources contaminated by agricultural runoff, heavy metals, and other pollutants [121]. Some NM have demonstrated the ability to effectively remove pesticide residues, fertilizers, and microbial pathogens, restoring the quality of water for irrigation and livestock use [122–124]. Integration of NM with smart irrigation systems, precisely controlling the delivery of water to crops based on real-time monitoring of soil moisture and plant needs [122, 123, 125]. This enhanced water-use efficiency not only conserves precious water resources but also reduces energy consumption and the carbon footprint associated with agricultural water management [126]. Additionally, the development of nano-enabled water-repellent coatings and membranes helps to minimize evaporative losses, further optimizing water utilization in arid and semi-arid regions [126–129]. Nanotechnology-enabled sensors can monitor the moisture levels in soil and activate irrigation systems selectively, thereby reducing water wastage to a minimum [130]. Graphene oxide, a type of nanomaterial, has been employed in water purification methods to eliminate impurities and disease-causing agents [131]. This helps to make irrigation water safer and reduces the likelihood of waterborne diseases damaging crops. As climate change and population growth heighten the pressure on global water supplies, the strategic deployment of nanomaterial-based technologies in agriculture holds the promise of a more sustainable, resilient, and water-secure future for food production.

### NM for precision nutrient management in agriculture

The strategic management of nutrients is a cornerstone of sustainable agriculture, ensuring optimal crop growth and productivity while minimizing environmental impacts. NM have emerged as powerful tools in revolutionizing nutrient management practices. NM are designed to encapsulate and deliver essential macro and micronutrients to plants in a highly targeted and controlled manner [33, 132, 133]. By controlling the release kinetics of nutrients, the uptake efficiency NM gets enhanced and minimize losses through leaching or volatilization, reducing the overall resource inputs required [33, 134, 135]. They are genetically modified to release nutrients in accordance with the plant's requirements, improving nutrient utilization and minimizing the negative effects of excessive fertilizers on the environment. Nanofertilizers are engineered to gradually break down and release nutrients, aligning with the way plants absorb minerals [135]. This helps reduce the amount of nutrients that are lost through leaching and runoff. Various nanoparticles have demonstrated the ability to solubilize and mobilize otherwise insoluble or inaccessible nutrients in the soil, making them available for plant absorption [136]. NM also serve as sensors, monitoring soil nutrient levels and plant physiological status in real-time, enabling precision fertilizer application tailored to the specific needs of crops [137]. As the agricultural sector grapples with the challenges of nutrient depletion, environmental pollution, and the imperative to improve resource use efficiency, the integration of nanomaterial-based technologies into nutrient management strategies holds immense potential to cultivate a more sustainable, productive, and environmentally responsible future for global food production.

### NM for addressing environmental stresses

NM alleviate the negative impacts of environmental pressures on soil and water resources. Superabsorbent nanoparticles effectively store moisture in drought-prone locations, hence aiding agricultural growth even during prolonged dry spells [138]. Some NM attach to excessive salts in saline soils, resulting in a decrease in soil salinity and an enhancement of the environment for crop growth [44, 139, 140]. Incorporating NM into soil and water management procedures not only improves agricultural output but also promotes the long-term viability of farming systems. Nanotechnology has the potential to address significant difficulties in modern agriculture by optimizing the utilization of natural resources and enhancing the ability of crops to withstand environmental pressures as depicted in Table 2.

**Table 2:** Impact of NM on soil and water management in agriculture

Category	Nanomaterial examples	Benefits in agriculture	Reference
Soil enhancement	Nano-clays, Nano-zeolites	Improve soil structure, aeration, and fertility; enhance water retention Improved seed germination, enhanced Nutrient absorption, increased crop yields	[117, 118]
Water management	Graphene oxide	Efficient water purification, improved irrigation systems	[50, 131, 141]
Nutrient management	Nanofertilizers	Controlled release of nutrients, reduced leaching and runoff	[6, 142, 143]
Environmental stress mitigation	Superabsorbent polymers	Retain moisture during droughts, reduce soil salinity	[144]
Smart agricultural technologies	Nanosensors, Nano-formulations	Precision farming, controlled nutrient delivery, early disease detection, environmental sustainability	[10, 75, 126]

## Enhancing crop growth and yield with nanotechnology

Nanotechnology offers innovative solutions to enhance crop growth and yield, addressing the pressing need for increased agricultural productivity in the context of a growing global population and limited arable land.

### Nanotechnology in seed treatment

Seed germination is a highly regulated process influenced by signaling molecules, including reactive oxygen species (ROS) and phytohormones. The generation of apoplastic ROS is essential for loosening the cell wall, which aids in water absorption and cell expansion [145]. Phytohormones like abscisic acid and gibberellins work in opposition to control germination and dormancy, while auxins help maintain dormancy [146]. ROS also play a role in modulating gene expression and hormone signaling related to germination. Zhang et al. [147] explain the breakdown of lipid and the leaching of bioactive substances by excessive ROS leading to oxidative damage, and impeding germination. Keeping ROS levels within an optimal range, referred to as the oxidative window, is vital for successful germination as explained in various studies [148, 149]. Seed priming is a traditional agricultural technique that enhances germination by hydrating seeds before sowing, using methods such as water soaking or physical treatments like ultraviolet light [150]. This preparation influences seed metabolism, aiding in reserve mobilization and cell wall loosening, resulting in better germination and stronger seedlings. Techniques may include hydro-priming, osmo-priming, or advanced methods like nano-priming, where nanoparticles enhance germination and stress tolerance. Nano-priming is an innovative seed priming technique that leverages the unique properties of nanoparticles to enhance seed germination, growth, and yield by improving stress resistance, water uptake, ROS regulation, and nutrient absorption, ultimately promoting robust plant development and productivity [151]. The nanoparticles serve as seed coatings, providing protection against pathogens and promoting faster growth by affecting gene expression and metabolic processes, thereby improving plants' resilience to both biotic and abiotic stressors. Nanotechnology has been effectively utilized in seed treatment to enhance germination rates and seedling growth as explained in various studies. [152–154]. Various authors have reported the use of nanoparticles such as silver and zinc oxide in the promotion of seed germination and protection against soil pathogens with improved plant health right from the initial stages of growth [155–157]. Studies indicate that nano-treated seeds exhibit faster germination, increased growth rates, and improved resilience against environmental stresses [158, 159].

### Nanofertilizers for enhanced nutrient efficiency

Nanofertilizers are an emerging technology designed to enhance nutrient efficiency in plants by delivering nutrients in the form of nanoparticles. Unlike conventional fertilizers, which often lead to nutrient runoff and environmental pollution, nanofertilizers offer a more targeted approach, releasing nutrients in a controlled manner that plants can readily absorb [135]. Nanoparticles, typically made from essential nutrients like nitrogen, phosphorus, and potassium, or micronutrients such as zinc and iron, are engineered to be more soluble and bioavailable than their traditional counterparts [4, 160, 161]. Nanofertilizers improve nutrient uptake by enhancing root-soil interactions, allowing plants to absorb nutrients more efficiently, thus reducing the need for frequent fertilizer applications. This leads to better crop yields, more sustainable agricultural practices, and reduced environmental impact due to minimized leaching and volatilization of nutrients. Nanofertilizers are formulated with biocompatible coatings that protect the nutrients from premature degradation and enhance their release based on the plant’s needs [162]. Nanofertilizers are combined with other growth-promoting substances, such as plant growth regulators, to further boost plant health and resilience. As agriculture faces increasing challenges like soil depletion and climate change, nanofertilizers present a promising solution for improving nutrient efficiency and sustainable food production. Thus nanofertilizers play a pivotal role in enhancing nutrient efficiency, offering a more controlled release of nutrients and reducing nutrient loss through leaching. These NM ensure that nutrients are available to plants at the right time and in the right quantities, thereby improving nutrient use efficiency and contributing to significant increases in crop yields. Research has demonstrated that nanofertilizers increase the bioavailability of nutrients, leading to healthier plants and higher agricultural productivity [142, 143, 163].

### Nano-biostimulants in crop development

Nano-biostimulants are an innovative approach to crop development that integrates nanotechnology with biostimulants to enhance plant growth, resilience, and productivity [164, 165]. These nano-formulations are designed to deliver active compounds, such as amino acids, peptides, humic substances, and microorganisms, at the nanoscale, significantly improving their bioavailability and efficacy [166]. By interacting with plant physiological processes at a cellular level, nano-biostimulants can enhance nutrient uptake, promote stress tolerance, and stimulate plant metabolism, leading to improved crop performance [164]. Nano-biostimulants, which include nano-encapsulated hormones and growth-promoting substances, have been found to stimulate plant growth and development [167, 168]. Additionally, their small size and high surface area allow for more efficient and targeted delivery, reducing the required dosage and minimizing environmental impact. Nano-bio stimulants also hold the potential to mitigating abiotic stresses like drought and salinity, further supporting sustainable agriculture practices as explained by Guerriero et al. [169] in tomato plant. Research highlights that nano-biostimulants represent a cutting-edge approach to enhancing crop development by combining nanotechnology with biostimulants to improve plant growth, resilience, and productivity. These nano-formulations increase the bioavailability and efficacy of active compounds, allowing for targeted delivery that enhances nutrient uptake and stress tolerance, ultimately leading to better crop performance while minimizing environmental impact. These NM enhance plant metabolism, improve nutrient uptake, and increase photosynthetic activity, resulting in stronger, more vigorous plants [168, 170]. The application of nano-biostimulants has been associated with increased crop yield and quality, showing potential in various agricultural systems [171].

### Role of nanotechnology in stress tolerance

The pivotal role is played by Nanotechnology in enhancing stress tolerance in plants by offering innovative solutions to mitigate the effects of various abiotic and biotic stressors. Through the use of nanoparticles, plants are fortified against challenges such as drought, salinity, extreme temperatures, and pathogen attacks [165, 169]. Nanoparticles are engineered to improve water retention in soil, regulate the uptake of essential nutrients, or enhance antioxidant enzyme activities within plants, enabling them to better cope with environmental stress. Metal-based nanoparticles like zinc oxide or silver nanoparticles have been shown to activate stress-response pathways and boost plant defense mechanisms [172]. NM are used to deliver stress-alleviating compounds, such as hormones or biostimulants, in a more controlled and efficient manner, ensuring timely response to adverse conditions. By improving the plant’s ability to manage oxidative stress and modulate physiological functions, nanotechnology offers a sustainable and promising approach to increase stress resilience, which is crucial in the face of climate change and its impact on global agriculture. Nanoparticles induce stress tolerance mechanisms in plants, helping them withstand drought, salinity, extreme temperatures, and pest attacks [173, 174]. This stress resilience contributes to more stable crop yields even under adverse environmental conditions [173, 175]. Nanotechnology significantly impacts agricultural practices, with a particular emphasis on enhancing crop growth and yield. Through various applications, from seed treatment to stress management, nanotechnology not only supports the growth of healthier and more robust crops but also promotes sustainable agricultural practices. The rising threats to food security from biotic stress and climate change have prompted a shift from synthetic pesticides to biopesticides, particularly in organic farming [176]. While microbial biopesticides offer a more sustainable solution, there is a need for comprehensive studies on the process parameters that affect their production.

## Safety, environmental concerns, and regulatory perspectives

The incorporation of nanotechnology in agriculture, while offering significant benefits, also raises important safety, environmental, and regulatory concerns. These concerns primarily revolve around the potential risks that NM might pose to human health, the environment, and the broader ecosystem.

### Safety concerns

The small size of nanoparticles allows them to penetrate biological membranes and access cells and tissues that larger particles cannot, making them highly effective in various agricultural and environmental applications [174, 177] However, this unique property also raises significant concerns about potential toxicity and long-term health impacts on agricultural workers, consumers, and the environment [12]. Research has demonstrated that certain NM exhibit toxic effects on plant cells, which can impair growth and development. Additionally, these nanoparticles can negatively affect beneficial soil microbes, disrupting microbial communities that are crucial for nutrient cycling and soil health. Aquatic life forms are also vulnerable, as nanoparticles can enter water bodies through runoff, potentially causing bioaccumulation and disrupting aquatic ecosystems, thereby impacting biodiversity and ecosystem services [178–180]. Several studies have suggested nanoparticles accumulate in plant tissues and the food chain, posing risks to human health and wildlife through bioaccumulation and biomagnification [181, 182]. These findings highlight the need for more comprehensive research to fully understand the long-term ecological and health implications of nanotechnology in agriculture and to develop guidelines for its safe use.

### Environmental impact

The environmental impact of NM has emerged as a critical concern, particularly due to the persistence of nanoparticles in ecosystems and their unknown long-term consequences [183, 184]. Unlike traditional materials, nanoparticles exhibit unique properties due to their small size and high surface area, which allows them to remain in the environment for extended periods without breaking down. This persistence raises significant concerns for soil health and water quality, as nanoparticles may alter the fundamental processes that sustain these ecosystems [185, 186]. Given the increasing use of NM in agriculture, industry, and consumer products, their release into the environment through runoff, leaching, or atmospheric deposition poses substantial ecological risks. Nanoparticles interact with environmental elements in ways that can lead to the formation of new pollutants or modify the behavior and toxicity of existing ones. Certain nanoparticles bind with contaminants already present in the soil or water, creating new compounds that are more harmful or more mobile than the original pollutants [187]. This can potentially exacerbate issues like groundwater contamination, soil degradation, or disruption of aquatic ecosystems. The work of Remédios [188] explored the ecological risks associated with nano-pesticides, highlighting that these materials can alter the bioavailability of nutrients and chemicals, affecting soil and water ecosystems in unpredictable ways. The increased mobility and reactivity of NM may also enhance the transport of pollutants across different environmental compartments, thereby complicating efforts to manage or remediate contamination. Another pressing concern is the potential for NM to bioaccumulate in organisms [189, 190]. Nanoparticles are taken up by plants, microorganisms, and animals, and as they move up the food chain, they may accumulate in higher trophic levels, including humans. The bioaccumulation of nanoparticles poses significant risks to biodiversity and ecosystem functioning, as these materials may interfere with biological processes at the cellular or molecular levels. Nanoparticles disrupt enzyme activities, cellular metabolism, and even genetic expression, as shown in Table 3, leading to adverse health effects in both wildlife and humans [191, 192]. The persistence of nanoparticles in the environment and their ability to enter the food chain underscore the need for comprehensive studies on their long-term effects. The potential environmental risks of NM necessitate thorough environmental impact assessments before they are applied on a large scale. Such assessments should examine not only the immediate ecological consequences but also the long-term effects of nanomaterial accumulation in different ecosystems.

**Table 3:** Key research in nanotechnology safety and environmental impact

Focus area	Study	Key findings	Reference
Genotoxicity	Toxicological effects of nanoparticles in plants	Nanoparticles alter gene expression, cause DNA damage, and lead to plant epigenetic variations Nanoparticles get accumulated in plant tissues, risking human and animal health	[198]
	Investigation of genotoxicity o in cucumber seedling	Photosynthetic pigments were affected by copper oxide nanoparticle applications Copper nanoparticles caused phytotoxicity of cucumber plants	[199]
	Genotoxicity assessment in wheat	Nano amino zinc could enter effortlessly into the cells and inhibit the normal cellular function	[200]
	Quantum dots genotoxicityin Allium cepa plants	The genotoxicity was observed to be dose-dependent, with the highest uptake and retention of cadmium occurring at the 50 nm concentration of Cadmium selenide quantum dots Concentrations as low as 25 nm of Cadmium selenide quantum dots were found to be cytotoxic 50 nm cdse qds were determined to be genotoxic to the plant	[201]
Ecological risks	Interaction of nanoparticles to soil microbial communities	Nanoparticles altered the structure of the soil microbial community Long-term exposure to nanoparticles decreased diversity of microbial communities	[202]
	Aluminum oxide nanoparticles toward Daphnia magna	H-Al_2_O_3_ nanoparticles were more toxic than α- Al_2_O_3_ nanoparticles in both short and long-term exposures Al_2_O_3_ nanoparticles pose no risk to aquatic organisms	[203]
	Adsorption of metals on aged microplastics in intensive mariculture areas	Aged Microplastic aggravate potential ecological risks of metals to marine organisms in mariculture area	[204]
	Graphene NM	At lower concentrations of tetrabromobisphenol-A), and graphene NM showed increase in toxic effects The risk quotient of tetrabromobisphenol-A towards the algae reduced in the presence of graphene NM	[205]
Regulatory challenges	Environmental protection	Nanoparticles are toxic when released into the environment, and study is needed to identify and describe environmental protection involving nanotechnology with respect to regulatory studies	[206]
	Regulatory aspects of NM used for agriculture	The increasing market of nanotechnology in conjunction with agriculture suggests a high boost in the economic viability of such nanoproducts The increased use of nanoproducts could be beneficial for meeting the growing demands for food However, their regulated use coupled with precautionary and safety measures is crucial to mitigate potential risks	[207]
	Nanotechnology in aquaculture	The potential effects of NM entering terrestrial habitats raise concerns due to their unknown interactions with soil ecosystems, which could affect plant and animal life Current risk assessment for NM is still nascent, with significant gaps in understanding their fate, transport, and effects in soil environments To effectively assess the risks to terrestrial organisms, there is a critical need for a comprehensive framework, such as the "Nanomix-soilrisk" framework, to evaluate the impact of nanoparticles in soil	[208]
	Nanotechnology-based agri-products	Nano-encapsulated nutrients/agrochemicals, antimicrobial agents, and food packaging Applicants must demonstrate the safe use of nano-based products for consumers and the environment Various countries employ different regulatory measures, including guidance and legislation, to ensure the safety of nano-based products in agriculture, feed, and food	[209]
	Ecotoxicological aspectsz of nanopesticides	Small particle sizes and high surface areas of nanopesticides can cause unintended (eco) toxicological effects on target and non-target species and the environment Limited understanding of the ecotoxicological effects and environmental fate of nanopesticides necessitates further research Stringent regulatory scrutiny and approval processes are required in most countries to assess the health and environmental risks before marketing nanopesticides	[210]

### Regulatory perspectives

Regulatory frameworks for nanotechnology in agriculture are still in developmental stages. These frameworks need to address the unique challenges posed by NM, including their manufacture, use, disposal, and lifecycle impacts [193, 194]. Policymakers are tasked with balancing the technological advancements in agriculture with potential health and environmental risks. As noted by various researchers, developing standardized methods for testing and evaluating the safety of NM is essential for regulatory approval and public acceptance [195, 196]. Given these concerns, there is a growing emphasis on research aimed at understanding the implications of nanotechnology. The development of 'green' nanotechnology, which emphasizes the use of environmentally friendly materials and processes, is particularly promising [197]. This approach seeks to mitigate the adverse effects of nanoparticles while harnessing their benefits for sustainable agriculture. The integration of safety, environmental, and regulatory considerations into the development and deployment of agricultural nanotechnologies is crucial for their responsible use. This integration ensures that while the agricultural sector can benefit from nanotechnology, it does not compromise human health or environmental integrity. Regulatory frameworks must be updated to ensure that the use of NM does not compromise environmental sustainability or public health. Only through rigorous testing and responsible application can the benefits of nanotechnology be realized without endangering the environment.

## NM in detection of pathogens and antioxidants in agriculture

NM have emerged as powerful tools in the detection of pathogens and antioxidants in agriculture, owing to their unique properties like high surface area, reactivity, and sensitivity. In the realm of pathogen detection, NM such as gold nanoparticles, carbon nanotubes, and quantum dots are extensively used to develop biosensors that provide rapid, accurate, and real-time monitoring of harmful microorganisms in crops and soil [211–213]. These nanosensors are capable of detecting low concentrations of pathogens due to their enhanced signal transduction capabilities, ensuring timely intervention and reducing crop losses. In the detection of antioxidants, NM play a crucial role in developing sensitive and selective sensors for monitoring the presence and concentration of bioactive compounds in agricultural products [211–213]. Nanoscale materials such as graphene and silver nanoparticles are used to fabricate electrochemical sensors that can accurately quantify antioxidants, providing valuable insights into the quality and nutritional value of fruits, vegetables, and grains [214, 215]. This is particularly beneficial for quality control and value addition in the agricultural supply chain. The integration of NM in pathogen and antioxidant detection not only promotes food safety and quality but also enhances the sustainability and efficiency of agricultural practices By harnessing the unique properties of NM, researchers and farmers achieve early detection and management of bacterial and fungal pathogens, as well as monitor the antioxidant levels in crops, which are vital for plant health and nutritional quality.

### Detection of bacterial and fungal pathogens

Detection of bacterial and fungal pathogens is crucial in various fields, including agriculture, medicine, and environmental science, as these microorganisms can cause significant harm to plants, humans, and ecosystems. Rapid, accurate, and sensitive detection methods are essential for effective disease management and prevention strategies. Traditional methods for detecting bacterial pathogens, such as culture-based techniques, are time-consuming and require specialized equipment [216]. However, modern molecular techniques like Polymerase Chain Reaction (PCR), quantitative PCR (qPCR), and Loop-Mediated Isothermal Amplification (LAMP) have revolutionized pathogen detection by offering rapid and specific identification of bacterial DNA [217, 218]. Nanotechnology offers innovative methods for rapid, sensitive, and accurate detection of bacterial and fungal pathogens in crops [219, 220]. In recent years, biosensors based on NM, such as gold nanoparticles, carbon nanotubes, and quantum dots, have been developed to enhance the sensitivity and specificity of pathogen detection [221–223]. These biosensors can detect low concentrations of bacterial pathogens in complex matrices, such as soil, water, and plant tissues, enabling early diagnosis and timely intervention [224, 225]. Detecting fungal pathogens is challenging due to their complex life cycles and morphological similarities [226, 227]. Traditional methods, like visual inspection and microscopic examination, are often inadequate for early detection and species differentiation. Molecular techniques, including PCR and Next-Generation Sequencing (NGS), have significantly improved the detection and identification of fungal pathogens [228, 229]. These methods allow for the detection of specific DNA sequences unique to fungal species, enabling precise identification. Immunological techniques, such as Enzyme-Linked Immunosorbent Assay (ELISA), are also employed for fungal pathogen detection. More recently, biosensors and portable devices incorporating NM and microfluidics have been developed to detect fungal pathogens in real-time, providing rapid, accurate, and on-site diagnostics.

### Monitoring of antioxidants

Nanotechnology also plays a significant role in monitoring antioxidant levels in agricultural produce. Antioxidants are crucial for plant defense against environmental stress and for enhancing the nutritional quality of the produce [230]. Nanoparticles such as zinc oxide and titanium dioxide have been employed in the development of biosensors that detect antioxidant [231, 232]. These sensors provide essential data on the health status of crops and guide agricultural practices to enhance crop resilience and nutritional output. The use of NM like silver nanoparticles and graphene-based nanocomposites has shown promising results in improving the sensitivity and selectivity of antioxidant detection [233]. These NM are incorporated into electrochemical sensors to enable the detection of low concentrations of antioxidants such as ascorbic acid, phenolic compounds, and flavonoids in fruits and vegetables. The high surface area and conductivity of NM enhance electron transfer rates, thereby improving sensor performance. This not only facilitates real-time monitoring of antioxidant levels but also supports the optimization of post-harvest storage conditions to maintain nutritional quality. The ability to precisely monitor antioxidant levels using nanotechnology also aids in the early detection of spoilage and quality degradation in agricultural produce [234, 235]. This information can be critical for supply chain management, ensuring that high-quality produce reaches consumers. As a result, the integration of nanotechnology in antioxidant monitoring contributes to sustainable agricultural practices, reduces food waste, and enhances the nutritional value of crops [236]. Overall, the convergence of nanotechnology and agricultural science offers innovative solutions for maintaining crop quality and promoting food security.

### Linking detection capabilities with sustainable practices

**Fig. 8:**
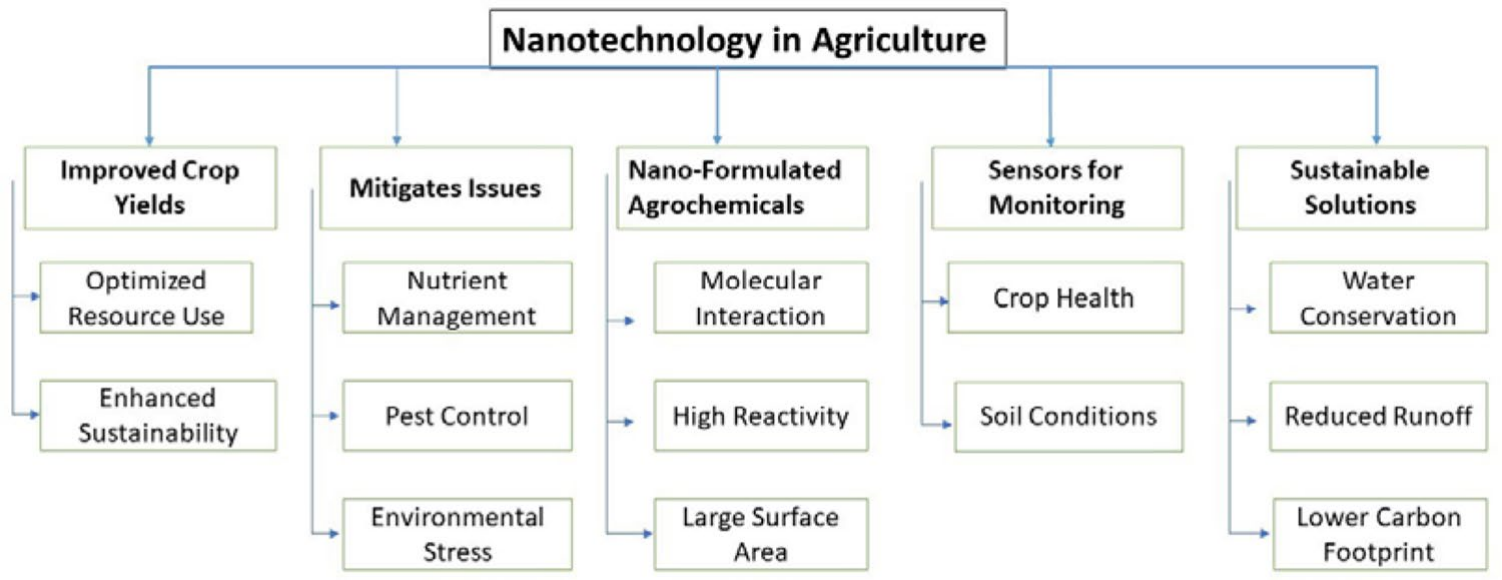
Nano technological advances in various Sustainable agricultural practices such Improved crops, Mitigates, sensors monitoring and sustainable solution

The integration of these nanotechnological innovations contributes to sustainable agricultural practices by reducing the need for chemical pesticides and fertilizers. Table 4 provides the overview of benefits of NM in agricultural with prime focus on the practice of utilizing them. The Early detection of pathogens allows for precise application of treatments, minimizing the environmental load of chemicals and enhancing the efficacy of pest management strategies [237, 238]. Similarly, understanding antioxidant levels in crops helps in managing crop health more effectively, ensuring high-quality produce without excessive chemical inputs. The overview of nanotechnological practices and their diverse applications in agriculture is depicted in Fig. 8.

**Table 4:** Table provides a snapshot of various NM and composites in agriculture, highlighting their preparation methods, utilization, and functional benefits

Nanomaterial/composite	Method of preparation	Use in agriculture	References
Silver nanoparticles	Reduction of silver ions in aqueous solution	Used as nanopesticides for their antimicrobial properties; enhance disease resistance in plants	[239, 240]
Nano-clays	Exfoliation and intercalation techniques	Improve soil water retention and nutrient delivery efficiency	[240, 241]
Carbon nanotubes	Chemical vapor deposition	Used in nanosensors to detect pollutants and soil nutrients	[242, 243]
Zinc oxide nanoparticles	Precipitation and sol–gel methods	Promote plant growth and protect against UV radiation; used in disease detection sensors	[75, 244, 245]
Nano-zeolites	Hydrothermal synthesis	Enhance soil nutrient retention and water holding capacity, thereby reducing water usage	[5, 118, 246, 247]
Graphene oxide	Modified Hummers’ method	Used in water purification systems to remove contaminants and improve water quality for irrigation	[141, 248–250]
Iron oxide nanoparticles	Coprecipitation	Used for soil remediation and as carriers for delivering plant micronutrients	[251–253]
Chitosan nanoparticles	Ionic gelation of chitosan with tripolyphosphate	Coating seeds to enhance germination rate and protect against fungal diseases	[254, 255]

## Future perspectives

The potential of nanotechnology in the detection of agricultural pathogens and antioxidants is set to grow. Advances in nanomaterial science are expected to lead to even more sensitive and selective detection systems, which could be integrated into a smart farming framework. This integration would allow real-time monitoring of crop health and environmental conditions, leading to an era of precision agriculture where decisions are data-driven and resource-efficient. The application of NM in detecting bacterial and fungal pathogens, as well as antioxidants, is transforming agricultural practices. By enabling early detection and precise monitoring, nanotechnology not only helps in managing crop health and enhancing yield but also aligns with sustainable agricultural goals. As research in this field continues to advance, the linkage of these technologies with everyday farming operations promises a future of increased agricultural productivity and sustainability.

### Challenges, limitations, and future directions

The integration of nanotechnology in agriculture faces significant challenges and limitations. Understanding these hurdles is crucial for harnessing the full potential of NM in enhancing agricultural productivity and sustainability.

### Technical challenges

One of the primary technical challenges is the mass production of NM. Scaling up the production of nanoparticles while maintaining their quality and functional properties is complex and costly. This challenge is compounded by the lack of comprehensive understanding of how these materials interact with diverse agricultural environments. Different soil types, crops, and environmental conditions react unpredictably to NM, making standardization difficult. The high cost of developing and deploying nanotechnology solutions also creates economic barriers, especially for small-scale farmers and those in developing regions, limiting access to these advanced technologies.

### Economic barriers

The high cost of developing and deploying nanotechnology solutions are unaffordable, especially for small-scale farmers and in developing countries. The initial investment for nanotechnology-based products is often higher than for traditional agricultural inputs. This economic barrier limits access to nanotechnology for many farmers, potentially widening the gap between large, technologically advanced farms and smaller, resource-poor farms.

### Regulatory and safety concerns

The regulatory landscape for NM is still in beginning. There is a pressing need for clear, science-based regulations that address the unique aspects of nanotechnology. This includes regulations concerning the use, disposal, and potential environmental impact of NM. There are significant safety concerns related to the exposure of farm workers and consumers to nanoparticles, which are not yet fully understood. Developing comprehensive safety protocols and ensuring compliance is a considerable challenge. Environmental and safety concerns also pose significant challenges. NM, due to their small size and high reactivity, have potential to accumulate in soil, water, and organisms, raising fears about toxicity, bioaccumulation, and long-term ecological impacts. The lack of comprehensive research on the long-term effects of NM on soil health, plant growth, and ecosystems further exacerbates these concerns. Regulatory frameworks governing the use of nanotechnology in agriculture are still underdeveloped, with no standardized guidelines to ensure safe application, disposal, and lifecycle management.

### Public perception and acceptance

Public perception of nanotechnology in agriculture influence its adoption and success. There is a degree of skepticism and fear regarding the use of such advanced technologies in food production. Concerns about the possible health impacts of consuming nano-enhanced foods could hinder consumer acceptance. Transparent communication and public engagement are essential to educate the public about the benefits and risks of agricultural nanotechnology.

## Future directions

While nanotechnology has shown promising results in laboratory settings, one of the biggest challenges moving forward will be scaling these innovations to meet the demands of global agriculture. Agriculture is a highly diverse field, with a wide range of crops, soils, and climatic conditions that may not respond uniformly to NM. Producing NM in the quantities needed for widespread agricultural use without losing their functional properties is a technical hurdle that researchers and industries must address. Developing cost-effective and scalable production methods for NM to improve their quality and functionality is critical for broader adoption. The focus should be on overcoming these challenges through continued research and development. Innovations in nanotechnology must go hand in hand with advancements in regulatory frameworks and safety protocols. Moreover, fostering collaboration among scientists, regulators, industry stakeholders, and the farming community is vital for the successful integration of nanotechnology in agriculture.

The development of cost-effective, scalable, and environmentally friendly nanotechnologies will play a crucial role. Emphasis should also be placed on creating inclusive technologies that are accessible to farmers worldwide, regardless of their economic status. While the path forward is fraught with challenges, the potential of nanotechnology to transform agriculture is immense. By addressing these challenges head-on and leveraging international cooperation and innovation, the future of agriculture can be significantly enhanced through the responsible and effective use of nanotechnology.

## Conclusion

The exploration of functional NM within agriculture has revealed their substantial transformative potential. These innovative materials offer promising solutions that revolutionize how we grow, protect, and enhance crop production. NM significantly enhance crop yields and sustainability by improving nutrient absorption and minimizing environmental impacts. From improving nutrient delivery through nanofertilizers to enhancing disease resistance with smart nanotechnology-driven solutions, the scope of benefits is extensive and profound. Innovations such as nano-priming and hydrogels are noted for boosting seed germination and drought resistance, while nanosensors enable real-time monitoring of soil and crop health. As the world grapples with the challenges of climate change, population growth, and environmental degradation, the role of sustainable practices becomes more crucial than ever. Nanotechnology, with its ability to provide targeted and efficient solutions, aligns perfectly with the global push towards sustainable agricultural practices.

However, the use of these materials also poses ecological risks, necessitating thorough assessments and regulatory frameworks to ensure their safe application. The future success of nanotechnology in agriculture needs innovative research, stringent safety evaluations, and robust regulatory frameworks. Fostering a positive public perception through transparency and engagement will be essential to the widespread adoption of these technologies. The responsible and effective use of nanotechnology in agriculture calls for a collaborative approach of scientists, policymakers, industry leaders, and farmers to ensure its benefits fully and equitably. Despite these advancements, significant research gaps remain concerning the long-term sustainability, scalability, and economic feasibility of nanomaterials in agriculture. These is an imperative need of comprehensive safety assessments and standardized protocols to guide responsible implementation, positioning nanotechnology as a transformative approach that enhances productivity and promotes ecological sustainability in farming practices. By continuing to innovate responsibly, we can harness the power of nanotechnology to not only enhance agricultural productivity but also contribute to a more sustainable and food-secure world.

## Data Availability

No datasets were generated or analysed during the current study.

## Abbreviations

NM: Nanomaterials
HA-NPs: Hydroxyapatite nanoparticles
LDHs: Layered double hydroxides
ZnO-NPs: Zinc oxide nanoparticles
NACs: Nano-agrochemicals
IPM: Integrated pest management
MIMO: Multiple input multiple output
RIS: Reflective intelligent surfaces
ROS: Reactive oxygen species
PCR: Polymerase chain reaction
qPCR: Quantitative PCR
LAMP: Loop-mediated isothermal amplification
NGS: Next-generation sequencing
ELISA: Enzyme-linked immunosorbent assay
